# Effectiveness and safety of adjunctive traditional Chinese medicine therapy for primary liver cancer patients: A protocol for systematic review and meta analysis

**DOI:** 10.1097/MD.0000000000021281

**Published:** 2020-07-31

**Authors:** Tingting Li, Xiaona Zhang, Shanshan Xu, Hongjie Cheng, Hong Zhang

**Affiliations:** Department of Spleen and Stomach, Fangshan Hospital, Beijing University of Chinese Medicine, Beijing, China.

**Keywords:** liver cancer, meta-analysis, protocol, systematic review, traditional Chinese medicine

## Abstract

**Background:**

Traditional Chinese medicine (TCM) has gradually drawn the attention of clinicians as an alternative for Primary Liver Cancer (PLC), is based on the theory of syndrome differentiation. However, owing to the lack of evidence-based medical evidence, the authors designed this protocol to evaluate TCM's effectiveness and safety.

**Methods:**

Seven electronic databases will be searched from inception to Aug. 2020. Two of us will independently identify randomized controlled trials, extract the data and assess the risk of bias. The meta-analysis will be conducted with the Review Manager 5.3 software according the heterogeneity of eligible studies. Further, we will use the grading of recommendations assessment, development, and evaluation to evaluate the evidence quality.

**Results:**

This study will demonstrate an evidence-based review of TCM for PLC.

**Conclusion:**

The study will provide clear evidence to assess the effectiveness and side effects of TCM for PLC.

**Trial registration number:**

PROSPERO CRD 42020173748.

## 1 Introduction

Primary liver cancer (PLC) is the sixth most common cancer in the world and ranks third in China.^[1]^ In 2005, the incidence of PLC in China was about 345,000, accounting for 50% of the world.^[1]^ PLC has a poor prognosis, and the 5-year survival rate reported between 1973 and 2007 is less than 12% in the United States.^[2]^ Due to the lack of sensitive screening tests, early PLC is currently difficult to diagnose. Therefore, at the time of diagnosis,^[3]^ only 30% to 40% of PLC patients can undergo potential treatment. Surgical resection, liver transplantation, local ablation and other treatment methods are only suitable for patients who retain liver function.^[4]^ However, most newly diagnosed PLC patients have reached advanced stages. For these patients with advanced PLC, treatment options are limited to palliative treatment, such as transcatheter arterial chemoembolization (TACE) or chemotherapy drugs.^[2]^ However, many patients are either not suitable for TACE, or conventional systemic cytotoxic chemotherapy^[5]^ is not effective. At the same time, the recurrence rate after PLC is high. Therefore, multidisciplinary treatment is essential for PLC patients. In China and some Southeast Asian countries, Chinese medicine has long been used to treat malignant tumors including PLC. With the development of Chinese herbal medicine technology, more and more Chinese medicine injections are used to treat PLC. Traditional Chinese medicines (TCM) are particularly suitable for elderly patients or advanced patients.

Studies have shown that TCM have multiple effects, such as anti-tumor angiogenesis, induction of tumor cell apoptosis, immune regulation and analgesia.^[6–9]^ TCM have become a popular anti-tumor therapy. Owing to the lack of evidence-based medical evidence, we designed this protocol to evaluate TCM's effectiveness and safety.

## 2 Methods

### 2.1 Protocol register

Our systematic review and meta-analysis protocol has been registered on the PROSPERO international prospective register of systematic reviews (ID = CRD 42020173748). We write the protocol were according to the Preferred Reporting Items for Systematic Review and Meta-Analysis Protocols guidelines.^[10]^ The final report will follow the recommendations of the PRISMA Extended Statement regarding the systematic review of the report's inclusion in the network meta-analysis of healthcare interventions.^[11]^

### 2.2 Ethics

Since the program does not require the recruitment of patients and the collection of personal information, no further ethical approval is required

### 2.3 Database search strategy

Respectively search all the articles published by the two authors by computer search and manual search. The searched databases include PubMed, EMBASE database, Cochrane Central Controlled Trials Register, China Biomedical Database, China National Knowledge Infrastructure, China Science Journals Database, Wanfang Database. All randomized controlled trials (RCT) on TCM for PLC will be searched to Aug. 2020. The specific search strategy will be formulated with a specific database. Among them, the author lists the search strategy of PubMed database (Table 1), and will be supplemented by manual search of relevant literature.

**Table 1:**
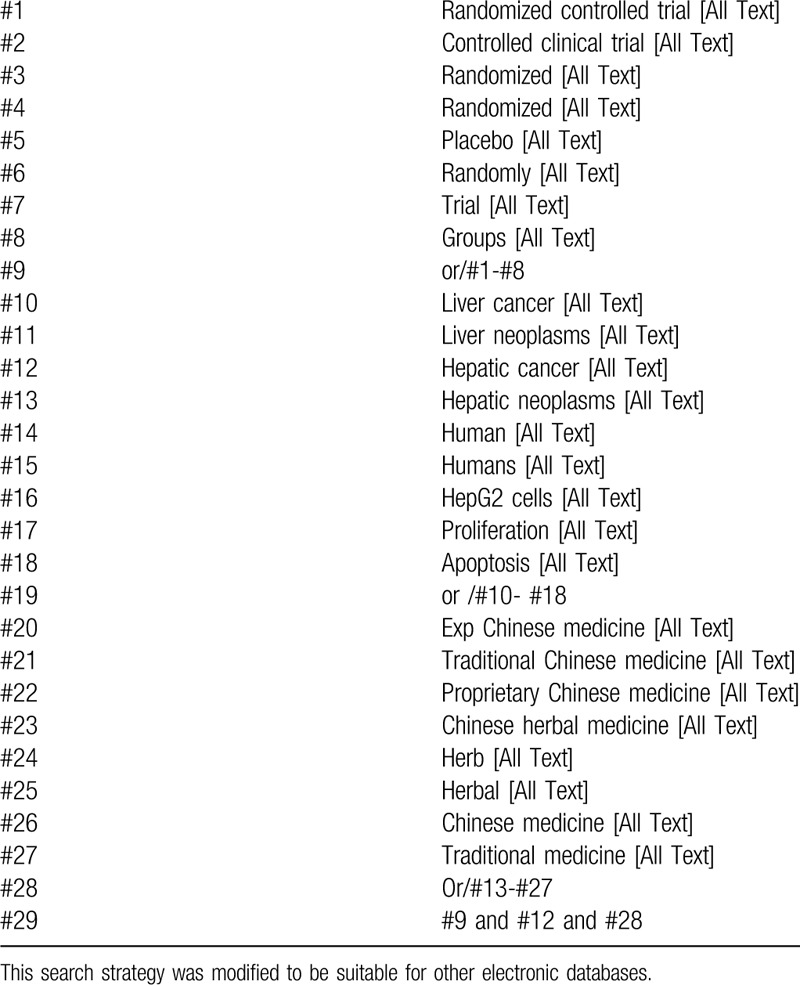
Search strategy for Cochrane library database.

We will also search for ongoing trial registrations on trial registration sites (such as the China Clinical Trial Registry and the National Institutes of Health resources) to obtain gray literature. At the same time, we also plan to manually search the reference list of comments captured by the initial search. There are no date restrictions, countries, publication status or publication year restrictions.

### 2.4 Eligibility criteria and elimination criteria

#### 2.4.1 Types of participants

Study of PLC patients confirmed by pathology, cytology, or imaging according to the National Comprehensive Cancer Network (NCCN) Clinical Guidelines for Liver Cancer.

#### 2.4.2 Types of interventions

##### 2.4.2.1 Observation group

TCM were used exclusively or in combination with other therapeutic methods. Types of Chinese medicines, combined use methods will be ignored.

##### 2.4.2.2 Control group

Other therapeutic methods (include the any other non-TCM treatment) or in combination with sham TCM.

#### 2.4.3 Types of outcome measures

##### 2.4.3.1 Main outcomes

Karnofsky Performance Scale score, quality of Life.

##### 2.4.3.2 Additional outcomes

Survival rate; improvement of clinical symptoms, such as abdominal pain and distension, fatigue, and loss of appetite; adverse events (AEs), including reduction in WBC and platelet counts.

#### 2.4.4 Type of study

Only Randomized controlled trials (RCTs) meets our requirements.

### 2.5 Study selection and data collection

**Figure 1:**
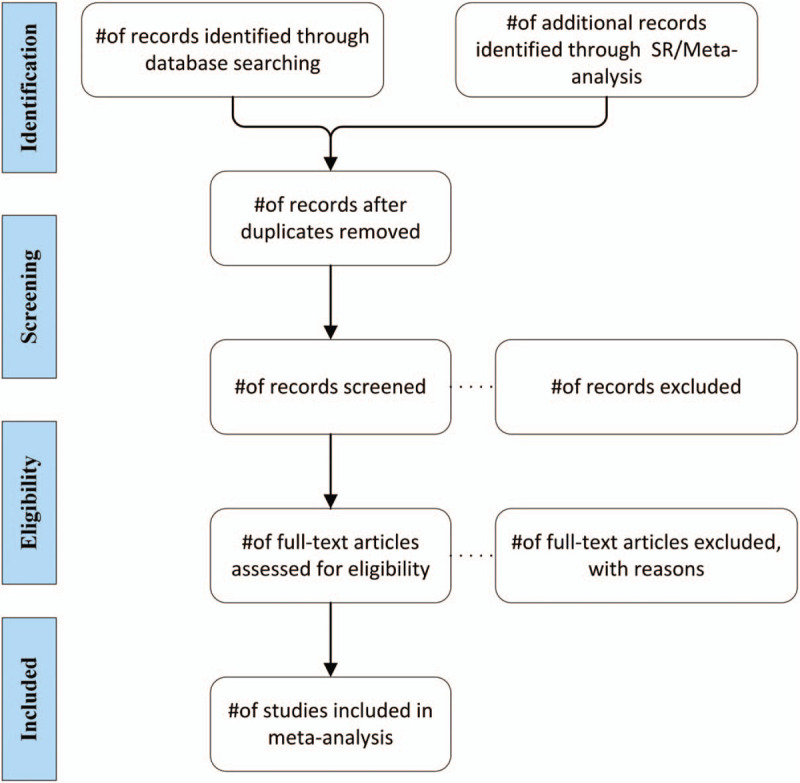
Study flow diagram, illustrate the process of studies selection.

The endnote X9 software will be used to manage all research. First, we will use this software to sort and sort out the documents, and exclude the repeated collection of documents. Second, the two studies will independently screen relevant studies that meet the inclusion criteria, based on the article's title, abstract, and keywords. Then, for uncertain research, we will download the full text for evaluation. This process will be completely completed by 2 researchers independently, and then cross-comparing their results. If the conclusions of the two evaluators are inconsistent, the differences can be resolved through discussion. If an agreement cannot be reached, we will seek the help of a third author for judgment and arbitration. In the list of excluded studies, we will report the reasons for the excluded studies in the full text review. A flowchart describing the search process will be included, including a reference list of all excluded studies. The suggested structure of the flowchart is shown in Figure 1.

Next, the 2 researchers will use the pre-designed Microsoft Excel data extraction form to independently extract the data in the study. The data items we plan to extract include:

1. Study characteristics (authors, journal, publication year, method of randomization, blinding method, etc.).
2. Participants (sample size, age, duration of disease, disease diagnostic criteria, etc.).
3. Intervention (Chinese medicine name, type of therapy, dosage forms, clinical doses, course of treatment, etc.).
4. Control (type of therapy, the course of treatment, dosage forms, clinical doses, etc.).
5. Outcomes (results, types of outcome measures, adverse events, etc.).

The two researchers will independently extract the data. If there is any objection, it can be discussed or negotiated with the third author. We will contact the original author via email to request any missing information, which may affect eligibility.

### 2.6 Dealing with missing data

We will obtain missing data by contacting the study authors, and discuss the reasons, degree, nature, and how to deal with missing data in each study. If data is still not available, we will only conduct a descriptive review of the study.

### 2.7 Literature quality assessment

Bias risk assessment included in the study Bias risk will be independently conducted by two scorers (Hua Zhen and Zhai Feng-Ting) based on the Cochrane bias risk tool. 35 It includes 7 specific areas:

(1) random sequence generation,
(2) allocation concealment,
(3) blindness of participants and personnel,
(4) blindness of result data,
(5) incomplete result data,
(6) selection Sex reports and
(7) other biases.

When evaluating the risk of bias in the study, if there is any disagreement, we will try to reach consensus between the 2 raters. If necessary, third-party assessors (Yang Guanlin or Zhang Zhe) will resolve their differences.

### 2.8 Statistical analysis methods

If no less than 2 RCTs are included in the study, we will conduct a paired meta-analysis. OR will be used to assess the magnitude of the impact of dichotomous variables, while the magnitude of the impact of continuous variables will be assessed using the mean difference. Because the included studies may cause heterogeneity in methodology, clinical, and statistical analysis, we will use a random effects model to synthesize the data.^[12]^ Heterogeneity is inevitable due to the method and clinical diversity that always exist in the meta-analysis. We will evaluate the heterogeneity of the study by calculating I2. The interpretation of I2 will be based on the threshold level proposed in the Cochrane collaboration. If there is significant heterogeneity that affects the results, we will perform subgroup analysis and meta-regression analysis to study potential impact modifiers, such as the participant's age, sample size, disease duration, treatment process, and study quality. The sensitive analysis will be used to check the stable of results. If the number of studies is greater than 10, we will also assess the publication bias of the included studies. As we all know, TCM research always involves the treatment of syndrome differentiation.^[13]^ However, this study only assesses whether traditional Chinese medicine is effective and safe for primary liver cancer. Therefore, this study does not conduct a meta-analysis of different TCM syndromes separately. In order to explore whether TCM syndromes cause heterogeneity in research results, we will make Subgroup analysis of TCM syndrome types.

### 2.9 Grading of recommendations assessment, development, and evaluation quality assessment

We will use grading GRADE to assess the quality of evidence and the strength of the main result recommendations.^[14,15]^ There are five factors that can reduce the quality of evidence: study limitations (risk of bias), inconsistency, discontinuity, publication bias, and imprecision. Accordingly, there are 3 factors that can improve the quality of evidence: confounding residuals, dose-response gradients, and large effects. The quality of evidence will be divided into four levels: very low, low, medium, and high. This step will be performed using GRADE profiler.

## 3 Discussion

Traditional Chinese medicine has been used to treat diseases for thousands of years, and it has been used in various diseases. However, the scientific nature of traditional Chinese medicine has been questioned. This study conducted a meta-analysis of TCM treatment for PLC, mainly to determine whether TCM is effective and safe for PLC. The results of this study will help clinicians and PLC patients choose whether to choose TCM for treatment. If the research results believe that TCM treatment of PLC has a greater benefit, then it will be the gospel of PLC patients. However, this study also has certain limitations:

1. There may be fewer RCT studies of TCM treatment of PLC, which will lead to a lower level of evidence for the study results and lack of reference value;
2. TCM treatment of diseases is mainly based on dialectical treatment.

However, due to the large individual differences in clinical syndromes, the study did not conduct separate meta-analysis based on different TCM syndromes, which may affect the external authenticity of the research results.

## Author contributions

**Conceptualization:** Tingting Li, Xiaona Zhang, Hongjie Cheng, Hong Zhang

**Data curation:** Tingting Li, Shanshan Xu

**Formal analysis:** Xiaona Zhang, Hongjie Cheng

**Methodology:** Hong Zhang

**Project administration:** Hongjie Cheng

**Supervision:** Hong Zhang

**Validation:** Hongjie Cheng

**Writing – original draft:** Tingting Li, Xiaona Zhang
